# Production of Mn-Ga Magnets

**DOI:** 10.3390/ma17040882

**Published:** 2024-02-14

**Authors:** Tetsuji Saito, Masahiro Tanaka, Daisuke Nishio-Hamane

**Affiliations:** 1 Graduate School of Engineering, Chiba Institute of Technology, Narashino 275-8588, Japan; 2 Institute for Solid State Physics, The University of Tokyo, Kashiwa 277-8581, Japan; hamane@issp.u-tokyo.ac.jp

**Keywords:** Mn-Ga alloys, melt spinning, spark plasma sintering, coercivity

## Abstract

Mn-based magnets are known to be a candidate for use as rare-earth-free magnets. In this study, Mn-Ga bulk magnets were successfully produced by hot pressing using the spark plasma sintering method on Mn-Ga powder prepared from rapidly solidified Mn-Ga melt-spun ribbons. When consolidated at 773 K and 873 K, the Mn-Ga bulk magnets had fine grains and exhibited high coercivity values. The origin of the high coercivity of the Mn-Ga bulk magnets was the existence of the D0_22_ phase. The Mn-Ga bulk magnet consolidated at 873 K exhibited the highest coercivity of 6.40 kOe.

## 1. Introduction

Nowadays, high-performance rare-earth magnets (Nd-Fe-B magnets) are applied to various advanced devices, including hard disk drives, electric vehicles, and medical equipment [1,2,3]. Because regulations on internal combustion engines have been enacted or proposed in many countries, the production of electric vehicles with high-performance rare-earth magnet motors has significantly increased [4]. Under such circumstances, the continuously growing demand for rare-earth elements, which constitute an integral part of high-performance rare-earth magnets, has raised severe concerns due to their prices and availability, together with the hazardous nature of the mining and smelting of such elements [5,6,7,8,9,10]. These concerns have led to the study of new magnetic materials composed of rare-earth-free elements.

The performance of rare-earth-free magnets is expected to be worse than that of rare-earth magnets, but the less-hazardous nature of rare-earth-free magnets could make them good substitutes for rare-earth magnets in some applications not demanding a good performance [11]. The prospective candidates for rare-earth-free magnets with high earth abundance are iron-based and manganese-based magnets. There have been many efforts to develop new iron-based magnets, and the L1_0_-FeNi intermetallic compound and α”-Fe_16_N_2_ nitrides have been identified as candidates for such magnets [12,13]. The current problem with the new iron-based magnets is the difficulty of their synthesis [14,15]. Until a new technique for their easy production is developed, these iron-based magnets are not considered highly promising.

On the other hand, manganese-based magnets have already been produced as commercial magnets [16]. More recently, manganese-based magnets, such as Mn-Ga magnets with a D0_22_ phase and Mn-Bi magnets with a low-temperature phase (LTP), have been the focus of attention as prospective replacements for rare-earth magnets [17,18,19,20,21,22,23,24,25,26]. It has been reported that the Mn-Ga alloys produced by melt spinning consist of a D0_22_ phase and exhibited high coercivity [27,28,29,30,31,32,33]. The advantage of Mn-Ga alloys is their high magneto-crystalline energy. Among the permanent magnets without rare-earth elements, Mn-Ga alloys possess the highest magneto-crystalline energy value, 2.6 MJ/m^3^. Thus, Mn-Ga alloys are expected to show high coercivity.

In this study, we seek the possibility of producing Mn-Ga bulk magnets. Nd-Fe-B melt-spun ribbons are known to be consolidated into bulk Nd-Fe-B magnets by hot pressing [34,35,36]. Mn-Ga magnets can be expected to be obtained from melt-spun ribbons similarly. The structures and magnetic properties of the Mn-Ga magnets produced by hot pressing using the spark plasma sintering (SPS) method from melt-spun ribbons are described here.

## 2. Materials and Methods

Rapidly quenched melt-spun ribbons of Mn_65_Ga_35_ alloy were prepared in a quartz crucible using single-roller melt spinning apparatus (NEV-A01, Nissin Giken, Saitama, Japan). Rapid quenching was carried out with argon gas using a copper wheel (v_s_ = 50 ms^−1^). Mn-Ga magnets were prepared from the rapidly quenched melt-spun ribbons. First, the rapidly quenched samples were mechanically comminuted into powders in an argon-filled glove box. The powders were then filled into carbon dies and consolidated in a vacuum at temperatures between 673 K and 973 K for 300 s using spark plasma sintering apparatus (Plasman, S. S. Alloy). A pressure of 100 MPa was applied during sintering.

The specimens were cooled to room temperature without applied pressure in the SPS chamber, and then pulled out from the carbon dies. After the surfaces of the specimens were cleaned using sandpaper, the properties of the specimens were examined. The density of the specimens was measured with Archimedes’ method using an electronic balance (GR-120, AND). The phases of the specimens were determined by X-ray diffraction (XRD) with Cu K_α_ radiation using an X-ray diffractometer (MiniFlex600, Rigaku, Tokyo, Japan). The microstructures of the specimens were examined using a transmission electron microscope (TEM) (JEM-2100, JEOL, Tokyo, Japan) after ion beam thinning using an Ion Slicer (EM-09100IS, JEOL). The hysteresis loops of the specimens were examined using a vibrating sample magnetometer (VSM) (BHV-525RSCM, Riken Denshi, Tokyo, Japan).

## 3. Results and Discussion

**Figure 1:**
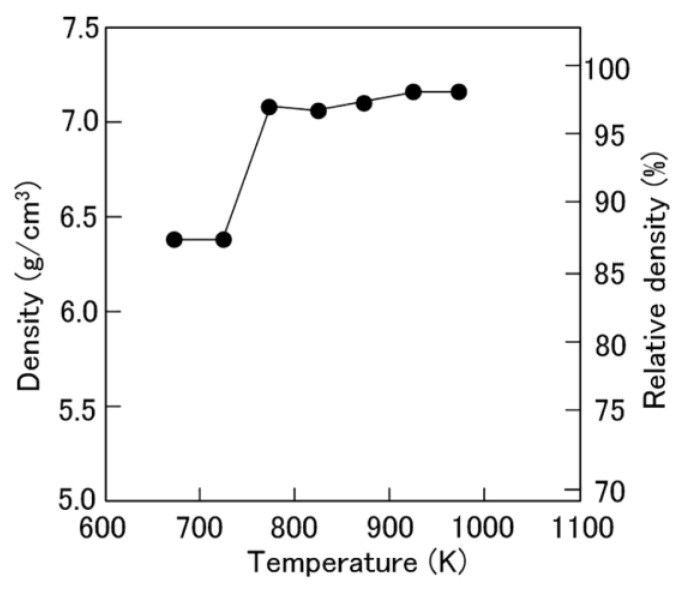
Dependence of the density and relative density of the Mn-Ga magnets magnet on the consolidation temperature.

Mn-Ga bulk magnets were successfully obtained from rapidly quenched Mn-Ga melt-spun ribbons by the SPS method. Figure 1 shows the dependence of the density and relative density of the Mn-Ga magnets on the consolidation temperature. The specimens had a density of about 6.4 g/cm^3^ when consolidated at 673 and 723 K. In contrast, the specimens had a high density of 7.1–7.2 g/cm^3^ when consolidated at 773 K or higher. These values represent approximately 97% of the ingot density. Since the typical density of Nd-Fe-B magnets produced by spark plasma sintering is 90–96% [29,30], the specimens consolidated at 773 K or higher had a sufficiently high density as magnets.

**Figure 2:**
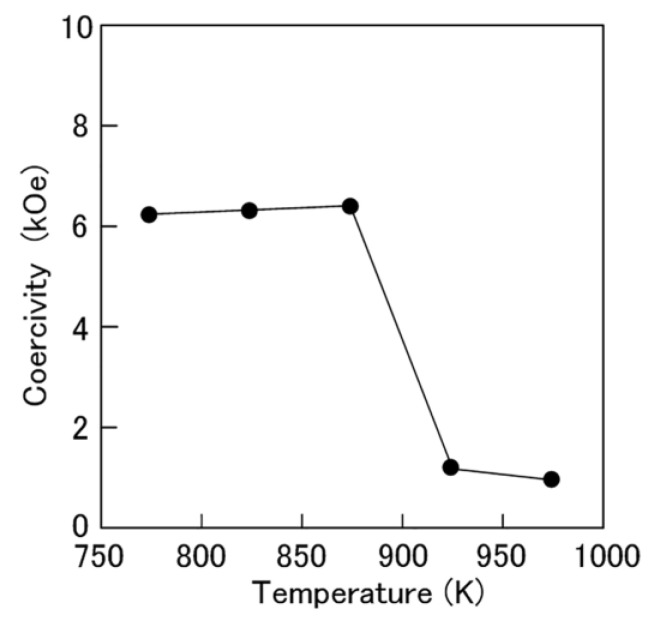
Dependence of the coercivity of the Mn-Ga magnets on the consolidation temperature.

Thus, the magnetic properties of the Mn-Ga magnets produced by consolidation at 773 K or higher were investigated. The consolidation temperature is a critical factor for not only the density of the magnets, but also the magnetic phase in the resultant Mn-Ga magnets. The dependence of the coercivity of the Mn-Ga magnets on the consolidation temperature is shown in Figure 2. The specimens showed high coercivity when consolidated at temperatures between 773 K and 873 K. In contrast, the specimens did not show high coercivity when consolidated at 923 K and higher. The Mn-Ga magnets consolidated at 873 K exhibited the maximum coercivity of 6.40 kOe in this experiment.

**Figure 3:**
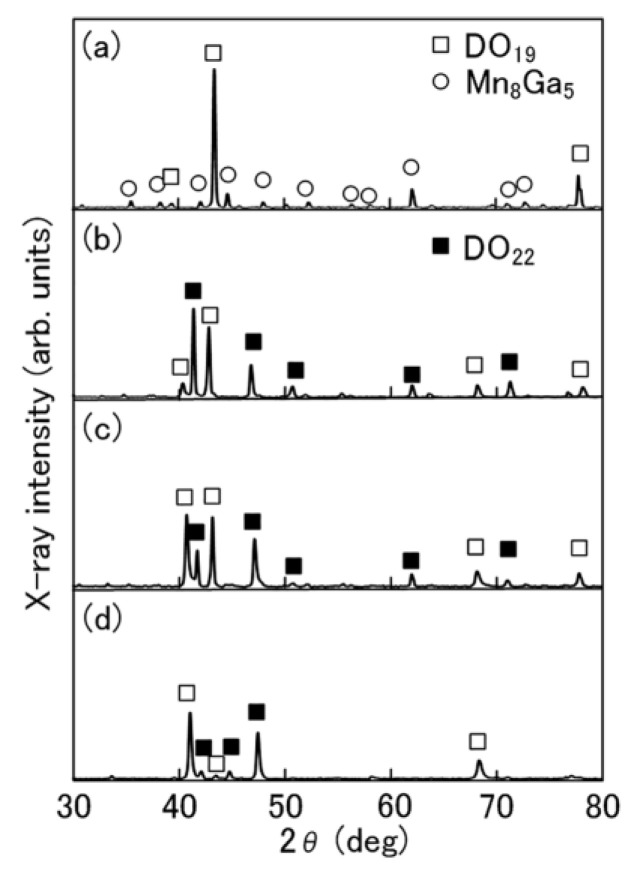
XRD patterns of (**a**) the Mn-Ga powder prepared from the Mn-Ga melt-spun ribbons and the Mn-Ga magnets consolidated at (**b**) 773 K, (**c**) 873 K, and (**d**) 973 K.

Among the Mn-Ga alloys, intermetallic compounds, such as the hexagonal D0_19_ phase, tetragonal L1_0_ phase, and tetragonal D0_22_ phase, have been investigated as magnetic materials [37]. Recently, it was found that the cubic P4_1_32 phase and cubic P4_2_32 phase exhibited high coercivity [38,39]. Figure 3 shows the XRD patterns of the Mn-Ga powder and the Mn-Ga magnets consolidated at 773 K, 873 K, and 973 K. The Mn_8_Ga_5_ and D0_19_ phases’ diffraction peaks were found in the Mn-Ga powder’s XRD pattern, suggesting that the Mn-Ga powder consisted of the Mn_8_Ga_5_ and D0_19_ phases. However, the D0_22_ and D0_19_ phases’ diffraction peaks were seen in the Mn-Ga magnets’ XRD patterns regardless of the consolidation temperature. This suggests that the Mn-Ga magnets consisted of the D0_22_ and D0_19_ phases. Although the Mn-Ga powder with the Mn_8_Ga_5_ and D0_19_ phases did not show high coercivity, the Mn-Ga magnets with the D0_22_ and D0_19_ phases exhibited high coercivity. Thus, the origin of high coercivity in the Mn-Ga magnets is considered to be the D0_22_ phase. The D0_22_ phase in the Mn-Ga magnets was formed due to heat exposure, while consolidating the Mn-Ga powder. Although the Mn-Ga magnets were mainly composed of the D0_22_ phase, they still contained some of the D0_19_ phase. Since the prolonged annealing of Mn-Ga melt-spun ribbons—for seven days, for example—could increase the amount of the D0_22_ phase [27], further optimization of the consolidation process can be expected to increase the amount of D0_22_ phase in the Mn-Ga magnets.

**Figure 4:**
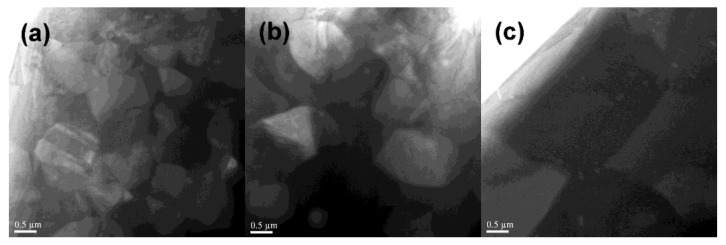
TEM images of the Mn-Ga magnets consolidated at (**a**) 773 K, (**b**) 873 K, and (**c**) 973 K.

Figure 4 shows TEM images of the Mn-Ga magnets consolidated at 773 K, 873 K, and 973 K. The specimens consolidated at 773 K had fine grains of around 0.5–1 μm in diameter, and the specimen consolidated at 873 K still consisted of fine grains of around 1–2 μm in diameter. On the other hand, the specimen consolidated at 973 K consisted of relatively large grains of around 5 μm in diameter. This indicates that grain growth was limited in the Mn-Ga magnet consolidated at 873 K, but significant grain growth occurred in the Mn-Ga magnet consolidated at 973 K. The Mn-Ga magnet consolidated between 773 K and 873 K consisted of fine grains of the D0_22_ phase, and thus, exhibited high coercivities. This confirms that the optimal consolidation temperature to produce high-coercivity Mn-Ga magnets is between 773 K and 873 K. 

**Figure 5:**
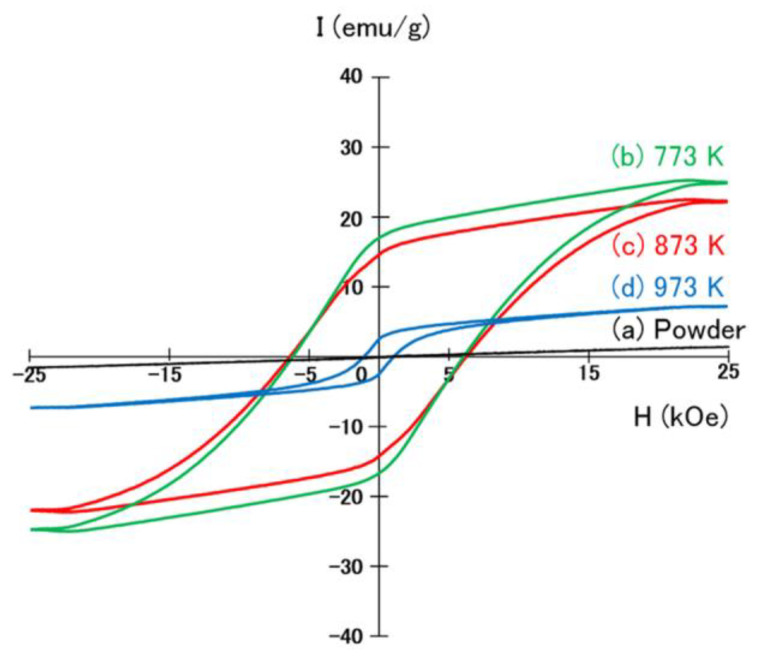
Hysteresis loops of (**a**) the Mn-Ga powder prepared from the Mn-Ga melt-spun ribbons and the Mn-Ga magnets consolidated at temperatures of (**b**) 773 K, (**c**) 873 K, and (**d**) 973 K.

Figure 5 shows the hysteresis loops of the Mn-Ga magnets consolidated at 773 K, 873 K, and 973 K, together with those of the Mn-Ga powder prepared from the rapidly quenched Mn-Ga melt-spun ribbons. The Mn-Ga powder did not show clear hysteresis, but the Mn-Ga magnets produced from the Mn-Ga powder exhibited hysteresis loops. The Mn-Ga magnet consolidated at 773 K showed a large hysteresis loop, with a coercivity of 6.20 kOe and a remanence of 17.9 emu/g, and the Mn-Ga magnet consolidated at 873 K also exhibited a large hysteresis loop, with a coercivity of 6.40 kOe and a remanence of 14.6 emu/g. The coercivity of 6.40 kOe achieved by the Mn-Ga magnet consolidated at 873 K was slightly smaller than that of the annealed Mn-Ga melt-spun ribbons (8 kOe) [27]. The Mn-Ga magnet consolidated at 973 K showed a relatively small hysteresis loop, with a coercivity of 1.05 kOe and a remanence of 2.55 emu/g. This suggests that the optimal consolidation temperatures to produce high-coercivity Mn-Ga magnets were between 773 K and 873 K.

There are several reports on the magnetic properties of Mn-Ga magnets. In the hot-compacted Mn-Ga magnets, a coercivity of 2.7 kOe was reported by T. Mix et al. [40]. In the cold-rolled Mn-Ga alloys, a high coercivity of 12.4 kOe was reported by S. Ener et al. [41] The high coercivity of the cold-rolled Mn-Ga alloy was achieved after magnetic annealing. The highest coercivity of 18.1 kOe was reported by J. Z. Wei et al. [42]. The Mn-Ga magnets with the highest coercivity have been produced by the long-term annealing of the Mn-Ga green compact, consolidated under a high pressure of 1.14 GPa. Therefore, magnetic or prolonged annealing can further improve the coercivity of the Mn-Ga magnets produced by the spark plasma sintering method.

## 4. Conclusions

Mn-Ga powder was prepared from rapidly quenched Mn-Ga melt-spun ribbons and consolidated into bulk magnets by the SPS method. It was found that the Mn-Ga bulk magnets had high densities of 7.1–7.2 g/cm^3^ when consolidated at 773 K or higher. Although the Mn-Ga powder did not show clear hysteresis, the Mn-Ga magnets exhibited hysteresis loops. It was found that the Mn-Ga magnets comprised the D0_22_ and D0_19_ phases. The Mn-Ga bulk magnets consolidated at 773 K and 873 K had fine grains and exhibited high coercivity values, whereas those consolidated at 973 K had coarse grains and did not show high coercivity. The Mn-Ga bulk magnet consolidated at 873 K exhibited a maximum coercivity value of 6.40 kOe. However, the remanence of the Mn-Ga bulk magnets is not yet comparable to that of the typical permanent magnets. Further studies are therefore necessary to increase the remanence value.

## Data Availability

Data are contained within the article.
